# Increased malaria incidence following irrigation practices in the Endorheic Rift Valley Basin of Sidama Region, Ethiopia

**DOI:** 10.1371/journal.pone.0284247

**Published:** 2023-04-25

**Authors:** Dawit Hawaria, Solomon Kibret

**Affiliations:** 1 School of Environmental Health, Hawassa University, Hawassa, Ethiopia; 2 California Department of Public Health, West Valley Mosquito and Vector Control District, Ontario, CA, United States of America; Noguchi Memorial Institute for Medical Research, University of Ghana, GHANA

## Abstract

**Background:**

Water resource development practice such as irrigation is key to ensuring economic growth and food security in developing countries. However, unintended public health problems such as malaria linked to such development projects have been a concern. This study aimed to determine the impact of irrigation on malaria incidence and vector mosquito abundance in southern Ethiopia.

**Methods:**

Eight years’ malaria morbidity data were extracted from the medical registers of health facilities in both irrigated and non-irrigated settings. Additionally, adult and larval malaria vector surveys were carried out in both irrigated and non-irrigated villages. The trend of malaria incidence, case distribution across age and sex, seasonality, parasite species proportion, and mosquito density were analyzed and compared between irrigated and non-irrigated villages.

**Results:**

The result showed that annual mean malaria incidence was 6.3 higher in the irrigated (95% CI: 0.7–33.6) than in the non-irrigated villages (95% CI: 1.2–20.6). Although a remarkable declining trend in malaria incidence was observed for four successive years (2013–2017), a significant resurgence between 2018 and 2020 was noted following the introduction of irrigation schemes. The densities of adult *Anopheles* mosquitoes were 15-fold higher in the irrigated compared to non-irrigated villages. Of the total potential mosquito-breeding habitats surveyed, the majority (93%) were from irrigated villages.

**Conclusion:**

Higher malaria incidence, adult *Anopheles* density, and mosquito-breeding habitat were recorded in the irrigated villages compared to non-irrigated villages. These observations have important implications for the effectiveness of existing malaria interventions. Environmental management could help reduce the breeding of malaria vector mosquitoes around irrigation schemes.

## Introduction

The global south has been experiencing extensive water resource development in recent years [1–4]. The primary aim of such development is to ensure the food security of the exponentially increasing population in the region. However, unintended public health problems of vector-borne diseases like malaria have been evidenced to be a concern around such development schemes. Previous studies indicated that land use changes related to water resources development such as irrigated agriculture have increased malaria transmission risks in some tropical areas by creating favorable conditions for parasites and their vectors [5–12].

Irrigation has not always been associated with increased malaria transmission. Some studies have reported that irrigation schemes either reduced or have no impact on malaria transmission and vector bionomics. For instance, Ijumba & Lindsay (2001) depicted a situation where a marked increase in *Anopheles* densities due to rice fields with no change in malaria incidence [7]. Another study in Kenya reported a lower prevalence of malaria in irrigated areas despite a 30–300 times higher abundance of the local malaria vector mosquitoes compared to non-irrigated [9]. In contrast, a study in Burkina Faso documented an average malaria prevalence rate ranging from 16–58% in an irrigated villages compared to 35–83% in non-irrigated villages [10]. Similarly, the study in Mali revealed a two-fold reduction in annual malaria incidence after the introduction of an irrigation scheme [11]. The proliferation of malaria vector breeding habitats and increases in adult mosquito density at irrigation schemes do not necessarily reflect a greater malaria transmission risk. Irrigation practice’s induced improvement in socio-economic status and housing conditions of nearby communities coupled with better health services and malaria interventions could help reduce vector-host contact, which eventually results in reduced disease transmissions in such development areas.

To promote economic growth and ensure food security, Ethiopia has embarked on extensive water resources development projects such as the construction of irrigation schemes on both large and small-scale [4]. Previous studies indicated that such development activities could lead to year-round malaria transmission and increased incidence in the respective localities [6, 12]. Those studies were conducted in limited areas, however, most malaria-prone areas of the country with irrigation practices still lack data on the effect of irrigation on malaria transmission. Even though Ethiopia is envisioning to embrace malaria elimination goals, the impact of such water infrastructure on public health, in terms of malaria transmission, is unclear.

The present study was conducted around the main Ethiopian rift in southern Ethiopia. Recently, small-scale irrigation practices by both local investors and farmers have been intensified to increase food production during the dry season, between November and March. The crops grown in the irrigation scheme includes papaya, banana, corn, and avocado. The malaria interventions like the distribution of bed-nets, indoor residual spray, and diagnosis followed by treatment have been exercised in the areas. However, data showing the effect of irrigation on malaria transmission in this region is missing. This study aimed to assess epidemiological and entomological indices of malaria transmission associated with irrigation practices in south Ethiopia.

## Methods and materials

### Study area

The study was conducted in Hawassa Zuria District (07° 01′ 54″ to 07° 50′ 36″ N and 38° 15′ 39″ to 38° 25′ 43″ E) of Sidama Region, about 275 km south of Addis Ababa, capital of Ethiopia. The district is transected to Hawassa lake, an endorheic basin in South Ethiopia. The lake’s watershed is located in the Ethiopian Rift Valley, between latitudes 6°4′45′′ N to 7°14′49′′ N and longitudes 38°16′34′′ E to 38°43′26′′ E. Lake Hawassa covers an area of 113 km^2^. It has a maximum depth of 10 meters and is situated at an elevation of 1,708 meters. The lake lacks an outflow, indicating that it must have a subterranean outlet [13].

Villages adjacent to the lake have started small-scale irrigation practices in recent years, since 2017. Due to the lake’s opportunity for irrigation, it attracted local investors who are involved in the production of the crop, including banana, corn, papaya, and avocado. The population size of the district was estimated to be 172,028 in 2020 [14]. The district covers around 22,643 hectares within the altitude range of 1700 to 1850 m above sea level.

### Site selection

Hawassa zuria has 24 kebeles, five of which are adjacent to lake Hawassa. For this study, four kebeles, two adjacent to the lake having irrigation practices and other two kebeles located at 2 to 3 km far away from the irrigation schemes, were selected. Malaria morbidity data was then collected from health facilities (two health centers and four health posts) of respective kebeles, for the years between 2013 and 2020.

For the entomological survey, two villages were selected from the irrigated and non-irrigated settings. Each village had between 100 and 150 households. The villages were delineated based on their convenience by location to transport logistics for mosquito collection. Apart from the difference in proximity to irrigation schemes, all villages share the same socioeconomic status and housing structure. From each village, five households were selected for adult mosquito collection.

### Morbidity data collection

Eight years of malaria data (2013–2020) were obtained from the health facilities in the study kebeles. Malaria cases were diagnosed using both microscope and rapid diagnostic test (RDT) by trained laboratory technicians. Test results were recorded in the malaria morbidity registration book, along with the patient’s name, age, gender, residence, date of diagnosis, and parasite species. The data were retrieved to Microsoft Excel and removed the patient’s name. The data were checked for completeness and incomplete case records were excluded from the analysis.

### Adult mosquito collection and processing

The entomological surveys were conducted both during the dry (February and March 2020) and wet (September and October 2020) seasons. The mosquito collection was made for 12 nights in each house during each collection season. Adult *Anopheles* mosquitoes were collected using the Centers for Disease Prevention and Control (CDC) light traps (Model: John W. Hock CDC Miniature Light trap 512, USA). The traps were set both indoors and outdoors. The traps set indoors were installed in a bedroom at about 1.5 m above the floor near the foot end of a person sleeping. Traps set outdoors were deployed between 1 and 5m away from an occupied house. The outdoor traps were installed at the same house where the indoor traps were set. Each trap was labeled to specific house identification and trapping location. The traps were kept operating from 18:00 to 06:00 hours. The next morning, after 06:00, the traps were collected and transported to the laboratory for species identification. In the laboratory, the specimens were retrieved from collection bags and sorted into species using morphological keys [18] under a microscope. The abdominal status of mosquitoes was determined as unfed, freshly fed, half-gravid, or gravid. The female *Anopheles* mosquito specimen was preserved individually in a labeled Eppendorf tube with desiccant for further laboratory analysis. The specimens were processed in the Environmental Health Laboratory at Hawassa University, Ethiopia.

### Larval survey

A mosquito larval survey was done to identify common *Anopheles* breeding habitats in the study area and the data was compared between irrigated and non-irrigated villages. The survey was conducted in the same villages where adult mosquito collection was made, in both irrigated and non-irrigated villages. Potential larval habitats were inspected within an estimated 1 km from the center of the study villages. The WHO standard larval survey procedure was followed using a standard dipper (350 mL, Bio Quip Products, Inc. California, USA) [15]. The presence and absence of *Anopheles* larvae in every stagnant water body were determined after making 10 dips or fewer depending on the size of the habitat.

### Data analysis

The malaria incidence rate was calculated as cases per 1,000 people at risk per year based on the catchment population and compared between irrigated and non-irrigated villages using the x^2^-test. Information of the population at risk was obtained from Hawassa Zuria district health office. Age and sex distributions, seasonality, and *Plasmodium* species composition over the study period were analyzed and compared between irrigated and non-irrigated villages. Both Microsoft Excel (Version 2016, Microsoft Corp, USA) and IBM SPSS version 20.0 (SPSS Inc., Chicago, IL, USA) statistical software packages were used for data analysis. In all statistical tests, a p-value at α less than 0.05 was considered significant. Adult mosquito density (number of mosquitoes per trap per night) was compared between the irrigated and non-irrigated villages. Density was calculated as: “D = n/trap-night” where ‘D’ is density for specific mosquito species and ‘n’ is the number of mosquitoes of respective species, and ‘trap-night’ represents the trapping night spent in all houses throughout the survey period.

## Results

### Malaria epidemiological profile

**Fig 1:**
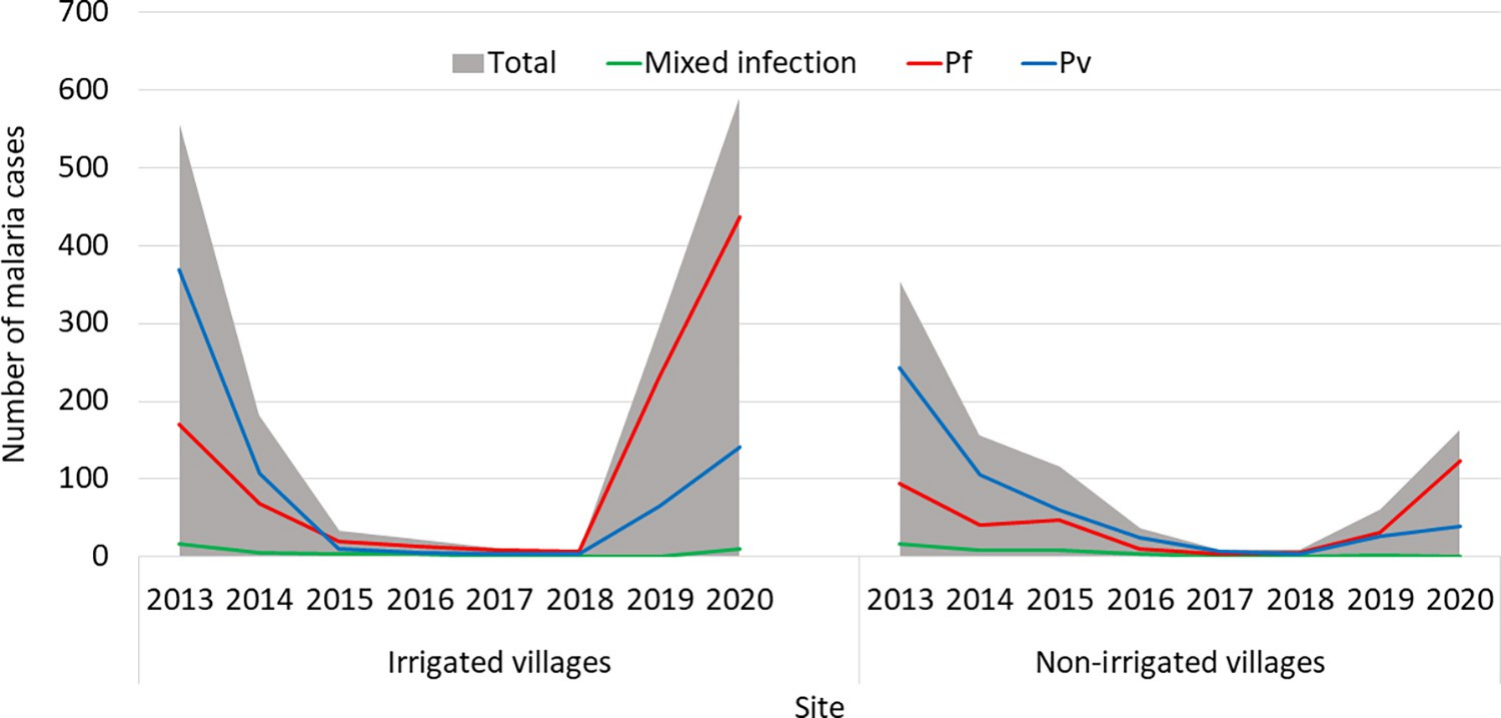
Annual number of malaria cases trend in the irrigated and non-irrigated villages, South Ethiopia, 2013–2020.

Between 2013 and 2020, a total of 2,609 confirmed malaria cases were recorded in the study area, of which 1,703 (65.3%) were from the irrigated villages and 906 (34.7%) were from the non-irrigated villages (Fig 1).

**Fig 2:**
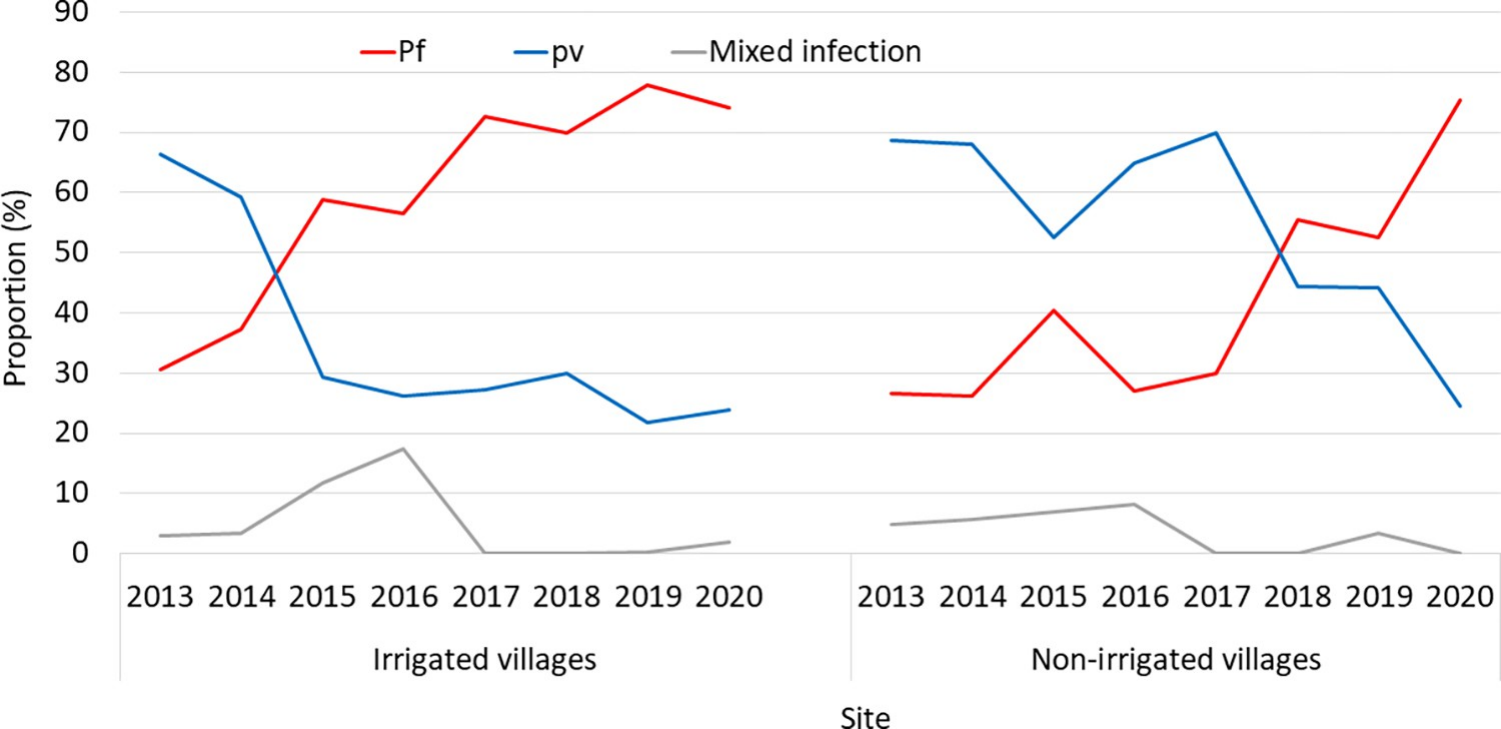
Proportion of *P*. *falciparum* (PF) and *P*. *vivax* (PV) parasites in the irrigated and non-irrigated villages, South Ethiopia, 2013–2020.

In the irrigated villages, *Plasmodium falciparum* (n = 955; 56.1%) was the predominant cause of malaria infection, followed by *P*. *vivax* (n = 705; 41.4%) and mixed infection (n = 43; 2.5%) (Fig 2). In contrast, *P*. *vivax* (n = 508; 56.5%) accounted for the majority of malaria cases in the non-irrigated villages while *P*. *falciparum* (n = 355; 39.2%) and mixed infections (n = 43; 4.3%) caused the remaining number of cases. Overall, *P*. *falciparum* was the dominant malaria parasite in the irrigated villages while *P*. *vivax* prevailed in the non-irrigated villages (Fig 2).

### Malaria incidence

**Fig 3:**
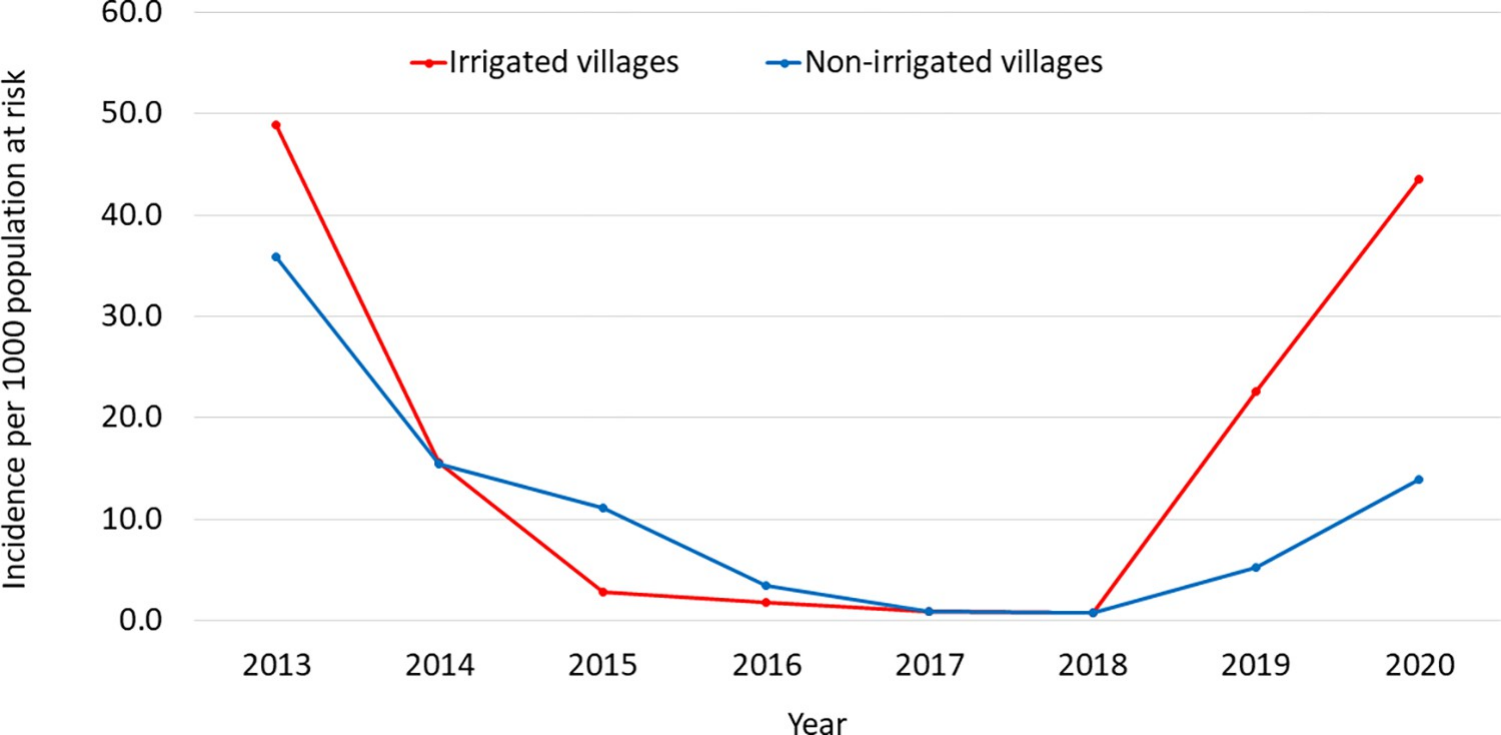
Annual malaria incidence in irrigated and non-irrigated villages, South Ethiopia, 2013–2020.

In the irrigated villages, the annual malaria incidence declined remarkably between 2013 (incidence = 49 cases per 1000 people) and 2017 (0.8 cases per 1000 people) (Fig 3). However, it dramatically increased between 2018 (22.6 cases per 1000 people at risk) and 2020 (43.6 cases per 1000 people at risk). A similar trend was also observed in non-irrigated villages with much lower annual malaria incidence figures. Overall, annual malaria incidence was 6.3 times higher in the irrigated villages (95% CI: 0.7–33.6) than in non-irrigated villages (95% CI: 1.2–20.6) during the study period (Fig 3).

### Malaria cases by sex and age

**Fig 4:**
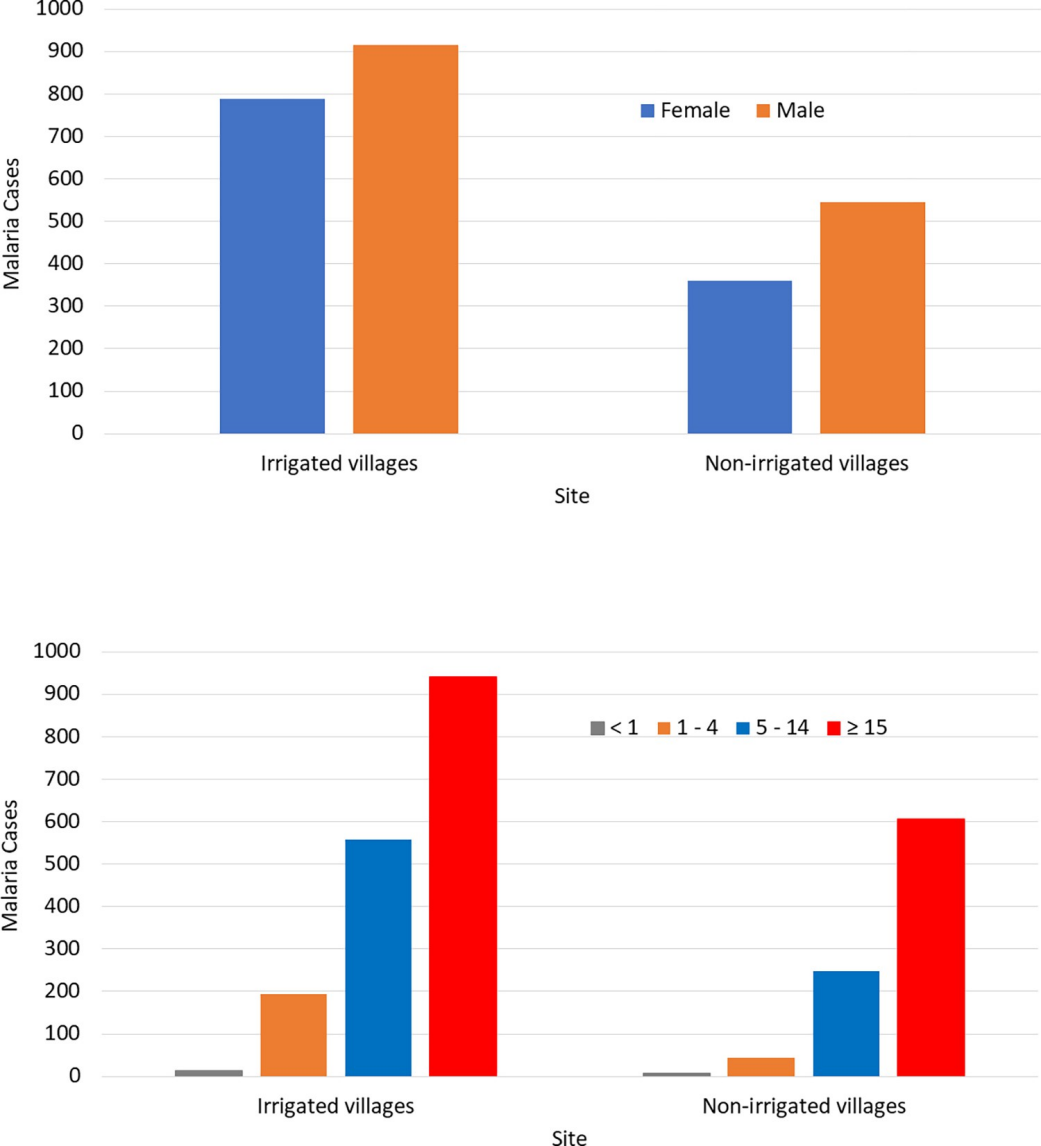
Malaria case distribution in the irrigated and non-irrigated villages, South Ethiopia, 2013–2020. (A) Malaria case distribution across sex. (B) Malaria case distribution across different age categories.

Of the total malaria cases in the irrigated villages, 915 (53.7%) were males and 788 (46.3%) were females. Likewise, more cases were reported in males (n = 545; 60.2%) than in females (n = 361; 39.8%)) in the non-irrigated villages. The data showed that males were more likely to get sick from malaria than females (χ^2^ = 9.91, d.f. = 1, *P* = 0.002) (Fig 4).

In irrigated villages, the majority of malaria cases were adults aged 15 years and above (n = 940;55.2%) followed by ages 5–14 years (n = 557; 32.7%). Similarly, two-thirds of malaria cases in the non-irrigated villages were male. In general, the malaria case number was found to be significantly higher in older male individuals in the study area (χ^2^ = 45.85, d.f. = 3, *P* < 0.0001) (Fig 4).

### Seasonal dynamics of malaria transmission

**Fig 5:**
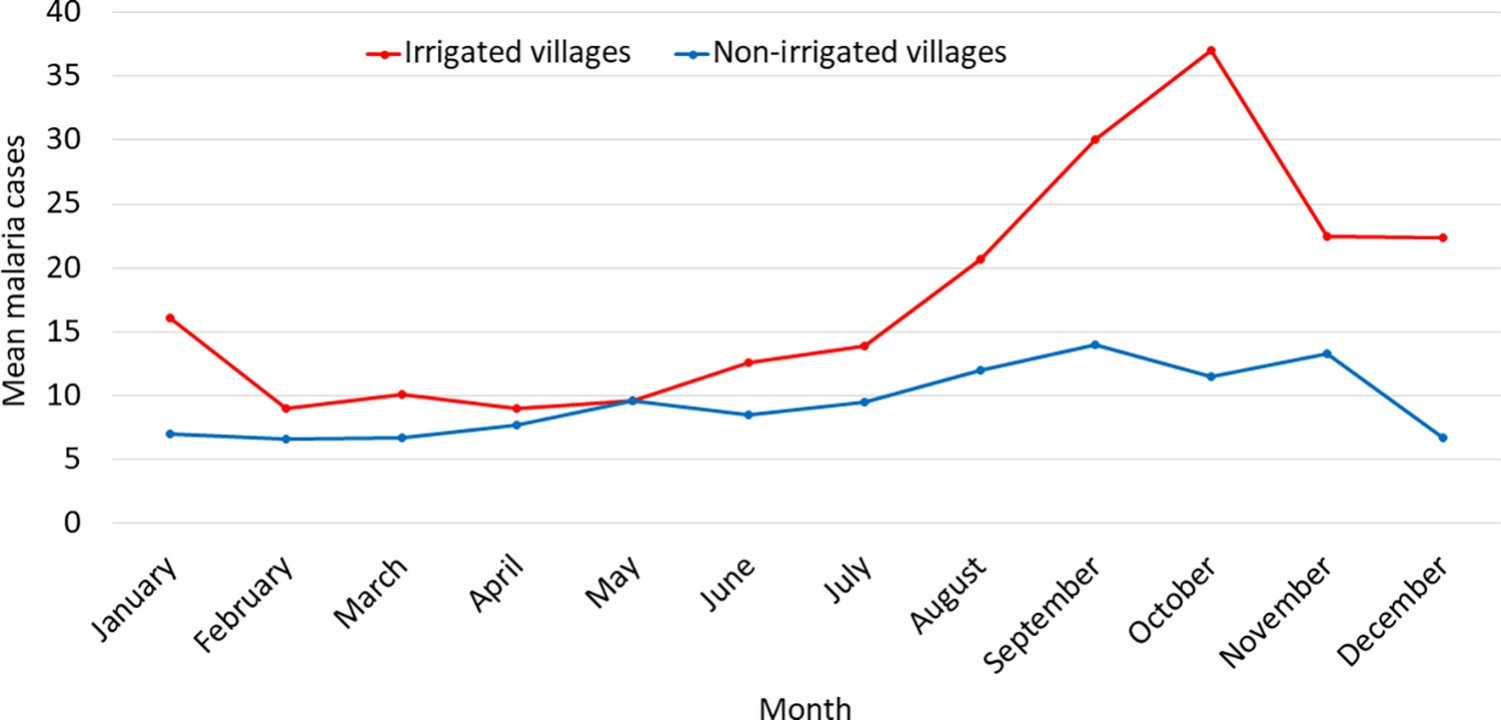
Seasonal dynamics of malaria transmission in the irrigated and non-irrigated villages, South Ethiopia, 2013–2020.

In the irrigated villages, a peak in malaria transmission was observed starting from September and extended up to January, the month of the dry season. The data showed that malaria transmission occurred across all months of the year in both irrigated and non-irrigated villages. However, significantly higher malaria cases were recorded in the irrigated than non-irrigated villages during the study period (χ^2^ = 72.37, d.f. = 11, *P* < 0.0001) (Fig 5).

### Adult *Anopheles* density

**Fig 6:**
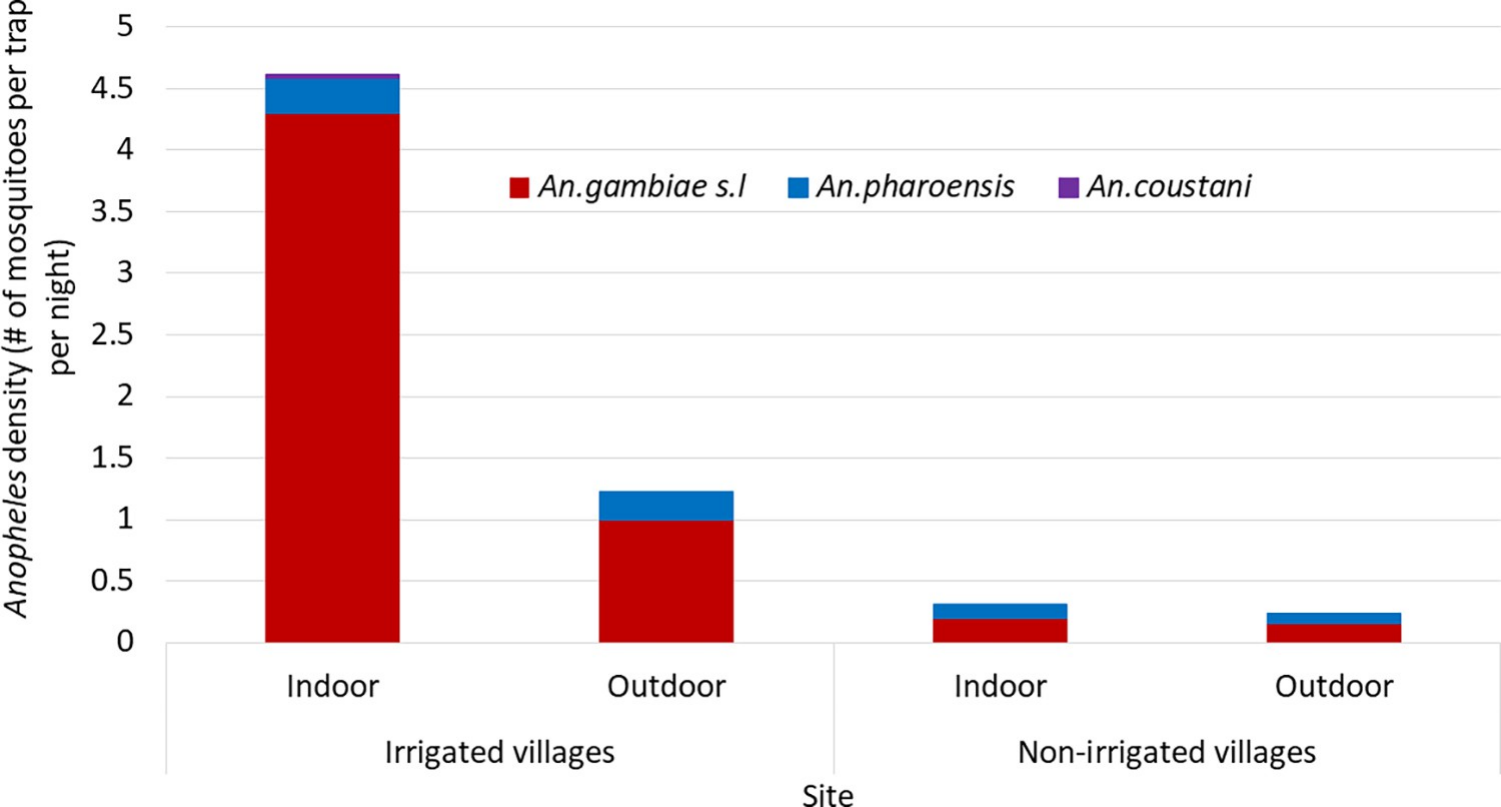
*Anopheles* mosquito species composition and density in irrigated and non-irrigated villages, South Ethiopia, 2020.

A total of 420 adult *Anopheles* were collected during the study period. The majority (93%) were collected from irrigated villages. Three *Anopheles* species were identified: *Anopheles gambiae* s.l, *An*. *pharoensis* and *An*. *coustani*. *Anopheles gambiae* s.l was the most predominant species in both the irrigated and non-irrigated villages. The densities of adult *Anopheles* were 15-fold higher in the irrigated villages than in the non-irrigated villages, with remarkably higher indoor density than outdoor. The densities of *An*. *gambiae* s.l was high during the wet season. In contrast, the densities of *An*. *pharoensis* were high during the dry season. Only small proportions of mosquitoes were found fed (Fig 6).

### *Anopheles* larvae breeding sites

A total of 58 potential mosquito-breeding habitats were surveyed, of which 54 (93%) were from irrigated villages. *Anopheles* mosquito larvae were found in 16 (29.6%) of surveyed habitats in the irrigated villages. Water-pit for irrigation, lakeshores, and road puddles were found to be common *Anopheles* larvae breeding habitats during both dry and wet seasons in the irrigated villages while none of the inspected habitats in the non-irrigated villages were found to be positive for *Anopheles* larvae (Table 1).

**Table 1:** Frequency of mosquito-breeding habitats surveyed in irrigated and non-irrigated villages, south Ethiopia, 2020.

Habitat types	Site			
	Irrigated villages		Non-irrigated villages	
	Frequency (%)	Positive for *Anopheles* mosquito larvae (%)	Frequency (%)	Positive for *Anopheles* mosquito larvae
**Water-pit for irrigation**	21(38.9)	9 (52.3)	-	-
**Lake shoreline**	12(22.2)	7 (47.7)	-	-
**Irrigation canal leakage**	11(20.4)	-	-	-
**Road puddle**	6(11.1)	-	2(50)	-
**Rain-pool**	4(7.4)	-	2(50)	-

## Discussions

The study indicated that malaria incidence was significantly higher in the irrigated villages compared to the non-irrigated villages. Though a successive decline in malaria incidence was observed between 2013 and 2017, recently, a remarkable increase in malaria incidence was detected in the study area which was highly pronounced in the irrigated villages. The mounting of malaria incidence was accompanied by irrigation practices in the area since 2017. Although the same malaria interventions by the government are in place in both settings, the enhanced malaria incidence in the irrigated villages might be attributed to irrigation practices. This study reaffirmed that the typical peak malaria season in Ethiopia runs from September to December [16–19]. However, peak transmission in irrigation villages extended to the month of dry season, January, with a higher number of cases compared to non-irrigated villages, which could be attributed to the continuous malaria vector availability due to the irrigation practice throughout most of the year. As a majority of parasite species in non-irrigated villages are *plasmodium vivax*, most cases could be a relapse rather than a new infection, which needs further parasite characterization investigation in the areas. The mass drug administration of primaquine, an anti-malaria that prevents relapse in *p*. *vivax* might help reduce the burden in the areas. However, because of its hemolytic toxicity, primaquine administration needs prior screening for primary factors to hemolysis like deficiency of glucose-6-phosphate dehydrogenase enzyme (G6PD) [20].

Malaria cases number in older male individuals were found to be higher in the study area, which is in agreement with previous studies elsewhere in Ethiopia, [17–19, 21–23]. Adult males are usually engaged in farming activities until the early period of the night during which they are more likely to encounter infective mosquito bites. Another potential explanation could be that bed nets are often used for younger children and mothers [24]. This study supported a current view that adults are emerging as a population domain that needs attention, which could be a challenge for the targeted malaria elimination goals [25–28]. As malaria interventions could induce change in malaria epidemiology, the control strategies need to be updated with transmission dynamics, like addressing LLINs to all age groups rather than targeting children and mothers alone.

This study reaffirmed the impact of irrigation in exacerbating malaria transmission risk as it was reported elsewhere including in Ethiopia [7, 12, 29]. Irrigation may create favorable conditions for the proliferation of mosquito-breeding sites, which enhance adult mosquito density and eventually elevate malaria transmission risk [30]. A 15-fold higher adult *Anopheles* density and frequent potential mosquito-breeding habitats in the irrigated villages indicate the increased malaria risk in the irrigated villages compared to the non-irrigated villages. Environmental parameters like temperature and humidity are important drivers of malaria transmission dynamics by influencing both the parasite and vector [31]. The crops in irrigated villages include papaya, banana, corn, and avocado, while seasonal corn in non-irrigated villages. Such land-cover differences may alter the microclimate in favor of parasite and vector, which could be one of the possible justifications for increased malaria incidence and its vector density in irrigated villages in this study. In contrast to our findings, some studies in Africa reported that irrigation had either no effect or reduced malaria transmission [7]. Improved socioeconomic status, better income, focused malaria interventions, and well-established health infrastructure were some of the explanations for reduced malaria transmission in the irrigation scheme settings [7, 32]. Irrigation practice could improve the income of the farmers which likely improves the socioeconomic status and housing conditions of nearby communities. Thus, improved economic status coupled with better health services and malaria interventions could help reduce vector-host contact, which eventually could result in reduced disease transmissions in the respective settings. The study indicated that *An*. *gambiae* s.l, presumably *An*. *arabiensis*, a primary malaria vector, predominantly feeds indoors than outdoors in both irrigated and non-irrigated villages. In the study area, the major vector control interventions like long-lasting insecticide nets (LLINs) distribution and indoor residual spray (IRS) were interrupted for the last three consecutive years, which could be a possible reason for high indoor density. As these interventions are effective for indoor feeding vectors, the finding suggests to resume and intensify the intervention strategies in the areas.

Lake Hawassa, an endorheic basin in southern Ethiopia, is a freshwater with irrigation potential for the surrounding communities. Previously, irrigation was not a common tradition in the area but presently, farmers and local investors have started extensive irrigation practices. The common irrigation modality in the area was surface irrigation with intermittent patterns. The sandy nature of the surrounding soil coupled with intermittent irrigation modality may help water not to stay long enough on the surface to support mosquito breeding. This could be a plausible justification for the absence of larvae on the irrigation runoffs and irrigation cannels’ leakages in the study area. In Kenya, a study reported that intermittent irrigation in the Mwea rice irrigation scheme resulted in lower mosquito larval densities [33]. On other hand, the less frequent mosquito-breeding habitats in the non-irrigated villages might be explained partly by the sandy nature of the soil at the locality. Water-pits for irrigation and lakeshores were the frequent malaria vector breeding sites encountered in the irrigated villages. Water-table around the lake is shallow and consequently, the surrounding communities practice constructing water-pit at their backyard to be pumped for irrigation. To document the dynamics of the *Anopheles* mosquito immature’s ecology in the study area, further longitudinal studies are warranted.

This study had its limitations. Firstly, malaria incidence was estimated using secondary data which sometimes has the issue of missing data, and active cases in the community also might be overlooked, which may lead to the underestimation of actual malaria incidence in the community. Secondly, it lacks microclimate data such as temperature and humidity which are an important malaria transmission dynamics drivers. They are sensitive to local land use and land cover change. Thirdly, for the interest of resource, the important entomological malaria transmission indices like entomological inoculation rate were also not addressed.

## Conclusion

Malaria incidence found to be higher in the irrigated villages than in the non-irrigated villages, which was supported by higher adult *Anopheles* density, and mosquito-breeding habitats, implying that irrigation practice in the area has been contributing to enhanced local malaria transmission risk. These observations have important implications for malaria interventions and communities’ awareness concerning irrigation. There is therefore a need for a tailored mosquito intervention strategy to control malaria transmission around irrigation areas. Further longitudinal studies of assessing entomological indicators of malaria transmission risk in relation to irrigation practice in the area are also needed. Additionally, routine surveillance and monitoring of both epidemiological and entomological information should also be in place to monitor disease dynamics along with irrigation development.

## Supporting information

S1 File (ZIP)

## Abbreviations

WRD: water resource development
LSM: larval source management
SPSS: Statistical Package for the Social Science
ACT: artemisinin-based combination therapy
IRS: indoor residual spray
LLINs: long-lasting insecticidal nets
ITNs: insecticide-treated nets
RDT: rapid diagnostic tests
EPHI: Ethiopian public health institute
SRHB: Sidama regional health bearua
